# A novel conjunctive microenvironment derived from human subcutaneous adipose tissue contributes to physiology of its superficial layer

**DOI:** 10.1186/s13287-021-02554-9

**Published:** 2021-08-28

**Authors:** Leandra Santos Baptista, Isis Côrtes, Bianca Montenegro, Cesar Claudio-da-Silva, Marielle Bouschbacher, Lara Jobeili, Celine Auxenfans, Dominique Sigaudo-Roussel

**Affiliations:** 1 Multidisciplinary Center for Biological Research (Numpex-Bio), Federal University of Rio de Janeiro (UFRJ) Xerém, Duque de Caxias, Rio de Janeiro 25245-390 Brazil; 2 Laboratory of Tissue Bioengineering, National Institute of Metrology, Quality and Technology (Inmetro), Duque de Caxias, Rio de Janeiro 25250-020 Brazil; 3 Post-Graduation Program in Biotechnology, National Institute of Metrology, Quality and Technology (Inmetro), Duque de Caxias, Rio de Janeiro 25250-020 Brazil; 4 Post-Graduation Program of Translational Biomedicine (Biotrans), Unigranrio, Campus I,, Duque de Caxias, Rio de Janeiro 25071-202 Brazil; 5 Plastic Surgery Service, Clementino Fraga Filho University Hospital (HUCFF), Federal University of Rio de Janeiro (UFRJ), Rio de Janeiro, Rio de Janeiro 21941-913 Brazil; 6 URGO Research Innovation and Development, Chenôve, France; 7 Hospital Civilian of Lyon, Hospital Edouard Herriot, 69003 Lyon, France; 8 Univ Lyon, Université Claude Bernard Lyon 1, CNRS, LBTI UMR5305, 69367 Lyon, France

**Keywords:** Human subcutaneous adipose tissue, Superficial microenvironment, Deeper microenvironment, Retinacula cutis microenvironment, Stromal vascular fraction, Adipose stromal/stem cells

## Abstract

**Background:**

In human subcutaneous adipose tissue, the superficial fascia distinguishes superficial and deep microenvironments showing extensions called retinacula cutis. The superficial subcutaneous adipose tissue has been described as hyperplastic and the deep subcutaneous adipose tissue as inflammatory. However, few studies have described stromal-vascular fraction (SVF) content and adipose-derived stromal/stem cells (ASCs) behavior derived from superficial and deep subcutaneous adipose tissue. In this study, we analyzed a third conjunctive microenvironment: the retinacula cutis superficialis derived from superficial subcutaneous adipose tissue.

**Methods:**

The samples of abdominal human subcutaneous adipose tissue were obtained during plastic aesthetic surgery in France (Declaration DC-2008-162) and Brazil (Protocol 145/09).

**Results:**

The SVF content was characterized in situ by immunofluorescence and ex vivo by flow cytometry revealing a high content of pre-adipocytes rather in superficial subcutaneous adipose tissue microenvironment. Adipogenic assays revealed higher percentage of lipid accumulation area in ASCs from superficial subcutaneous adipose tissue compared with retinacula cutis superficialis (*p* < 0.0001) and deep subcutaneous adipose tissue (*p* < 0.0001). The high adipogenic potential of superficial subcutaneous adipose tissue was corroborated by an up-regulation of adipocyte fatty acid-binding protein (FABP4) compared with retinacula cutis superficialis (*p* < 0.0001) and deep subcutaneous adipose tissue (*p* < 0.0001) and of C/EBPα (CCAAT/enhancer-binding protein alpha) compared with retinacula cutis superficialis (*p* < 0.0001) and deep subcutaneous adipose tissue (*p* < 0.0001) microenvironments. Curiously, ASCs from retinacula cutis superficialis showed a higher level of adiponectin receptor gene compared with superficial subcutaneous adipose tissue (*p* = 0.0409), widely known as an anti-inflammatory hormone. Non-induced ASCs from retinacula cutis superficialis showed higher secretion of human vascular endothelial growth factor (VEGF), compared with superficial (*p* = 0.0485) and deep (*p* = 0.0112) subcutaneous adipose tissue and with adipogenic-induced ASCs from superficial (*p* = 0.0175) and deep (*p* = 0.0328) subcutaneous adipose tissue. Furthermore, ASCs from retinacula cutis superficialis showed higher secretion of Chemokine (C–C motif) ligand 5 (CCL5) compared with non-induced (*p* = 0.0029) and induced (*p* = 0.0089) superficial subcutaneous adipose tissue.

**Conclusions:**

This study highlights the contribution to ASCs from retinacula cutis superficialis in their angiogenic property previously described for the whole superficial subcutaneous adipose tissue besides supporting its adipogenic potential for superficial subcutaneous adipose tissue.

## Background

White adipose tissue develops in distinct regions of the body called depots. The largest adipose tissue depots are abdominal subcutaneous white adipose tissue under the skin and visceral white adipose tissue that surrounds internal organs [1]. In rats, subcutaneous adipose tissue (SAT) arises from superficial fascia [2] expanding along multiple layers [3, 4]. In humans, the superficial fascia distinguishes two main layers in SAT: superficial and deep [5]. The superficial fascia has extensions called retinacula cutis (RC). The RC is formed by loosely interlaced collagen fibers that surround the adipocytic lobes [6]. The RC works as a structural element of the RC related to facial skin flaccidity or in relation to cellulite in other parts of the body [7–9]. However, until now, there is no published study related to the cellular components and biological significance of RC in humans.

The deep SAT (dSAT) is related to the obesity-associated complications similarly to the visceral adipose tissue. Recently, Lee and collaborators described a significant correlation between serum levels of inflammatory cytokines and adipokines with the sum of dSAT area [10]. On the other hand, two independent studies [11, 12] revealed a higher adipogenic potential for adipocyte progenitor cells isolated from the superficial SAT (sSAT). Furthermore, gene expression analysis revealed several metabolic and anti-inflammatory genes, including adiponectin preferentially expressed in sSAT, whereas inflammatory genes are over-expressed in dSAT in human obese samples [13].

Recently, Di Taranto et al. described some remarkable differences in gene expression of stromal-vascular fraction (SVF) showing a higher VEGFA expression in SVF from sSAT and a preferably location of CD34 positive cells (pre-adipocytes) surrounding blood vessels compared to dSAT in normal weight samples [14]. An additional study showed no differences in the percentage of CD146 positive cells (mesenchymal stem cells) for both layers of SAT [10]. We can postulate that the dSAT has an inflammatory profile, while sSAT has a higher adipogenic potential. Furthermore, the RC, from superficial fascia, should not be neglected since it has already been proved an origin for SAT from superficial fascia in a rat in vivo model [2]. The RC could represent a relevant microenvironment of adipose-derived stromal cells (ASCs) in human SAT.

The aim of this study was to decipher SVF progenitor subpopulations within the three-specific microenvironment: sSAT, dSAT and superficial RC (sRC) with a specific focus on adipose-derived stromal/stem cells (ASCs) involved in tissue remodeling and regenerative medicine approaches [15–17]. We chose to analyze only the RC from the superficial layer due to the adipogenic properties already described in the scientific literature for sSAT. ASCs from these three distinct microenvironments were characterized and challenged in terms of adipogenic potential and secretion of growth factors and cytokines. To the best of our knowledge, this is the first time in the scientific literature that ASCs are also described in human sRC.

## Methods

### Subcutaneous adipose tissue harvesting

**Fig. 1:**
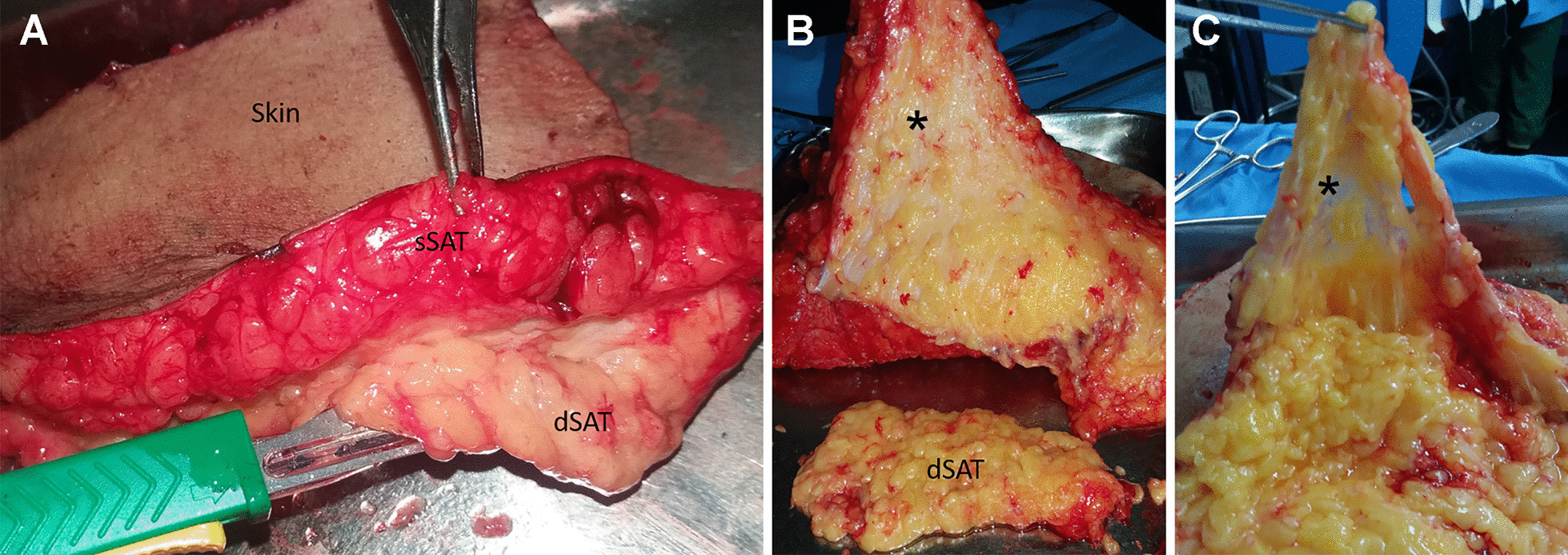
Macroscopic aspects of human SAT. **A** Representative image showing the skin, sSAT and dSAT. **B** Dichotomy of sSAT layer revealing the superficial fascia (asterisk), a dense conjunctive tissue located between sSAT and dSAT layers. **C** The exposure of the sSAT layer revealed the sRC (asterisk), a loose connective tissue located inside sSAT. *SAT* subcutaneous adipose tissue, *sRC* superficial retinacula cutis, *sSAT* superficial SAT, *dSAT* deep SAT

Abdominal SAT was obtained during plastic aesthetics in France (Declaration no. DC-2008-162 delivered to the Cell and Tissue Bank of Hospices Civils de Lyon), (*n* = 3, BMI: 25.1 ± 4) and Brazil (Research Ethics Committee of the Clementino Fraga Filho University Hospital, Federal University of Rio de Janeiro, Protocol 145/09), (*n* = 6, BMI: 25.4 ± 2.4), and informed consent was obtained from all donors included in the study. To obtain the sSAT and dSAT, we firstly identified the superficial fascia, a dense connective tissue. Both layers had well-defined lobes (Fig. 1A) and are separated by the superficial fascia (Fig. 1B). Subsequently, the dSAT was lifted using surgical tweezers and dissected using a scalpel (Fig. 1B), followed by the removal of the superficial fascia to ensure the absence of contamination between sSAT and dSAT. In this study, the sRC was dissected only from sSAT, easily identified due to its fibrous characteristics of a loose connective tissue (Fig. 1C). The samples were stored at 4 °C after the surgery, and the isolation of SVF was performed within 18 h.

### Immunofluorescence analysis

The whole SAT was cut off by a scalpel into small pieces containing all layers and immediately submerged in OCT compound (Euromedex, Strasbourg, France). The samples were maintained at − 70 °C freezer. Sections of 16 μm were prepared using the cryostat (Leica CM1520) and collected onto apex adhesive slides (Leica). The slides were maintained at − 20 °C freezer until immunostaining protocol. For immunofluorescence analysis, cryosections were incubated with a solution composed of acetone/methanol (1/1, v/v) for 10 min at − 20 °C followed by washes in phosphate buffered saline (PBS) containing 0,3% of Triton X-100 (PBS-T). Non-specific marking was blocked using PBS-T solution containing 1% of bovine serum albumin (BSA, Sigma, St Quentin Fallavier, France) for 1 h at room temperature. The following anti-human primary antibodies diluted in 1% BSA PBS-T were incubated overnight at 4 °C: Pref-1 anti-goat (R&D Systems, 1:100), CD34 (Progen, 1:200), CD34 (Abcam, 1:100), CD31 (Chemicon, 1:100) and CD146 (Abcam, 1:100). After washing in 0,3% PBS-T, secondary antibody staining was performed using Alexa-488 anti-mouse and anti-goat (Invitrogen, 1:1000) and Alexa-568 anti-mouse and anti-rabbit (1:500) for 1 h at room temperature together with nuclear staining (Hoetcsh, 1:1000). Sections were washed with 0,3% PBS-T, mounted in Fluorescence Mounting Medium (Dako), and examined under fluorescence microscope. Non-specific binding of secondary antibodies was monitored by carrying out the immune reaction in the absence of the primary antibody. We performed four independent experiments (*n* = 4) of three donors (*n* = 3).

### Cell isolation and culture

The sSAT, sRC and dSAT from SAT were cut into small fragments and incubated with 1 mg/ml collagenase type I (Sigma) in a water bath at 37 °C for 15 min. After digestion, the tissues were centrifuged at 400 g at room temperature for 15 min to eliminate the mature adipocytes. To eliminate tissue debris, the pellet was filtered in a 100 μm mesh filter.

For cell counting, the cell suspension was diluted in dye Turk liquid in a 1:100 ratio (10 µL of cell suspension in 90 $\mu$L of dye Turk liquid) and counted in a Neubauer chamber, so that all nucleated cells stained in blue and red cells are not seen by the action of acetic acid. After SVF quantification, cell suspension was divided for flow cytometry analysis and cell culture. To obtain the adherent cells—termed as adipose derived stromal/stem cells (ASCs), cell suspension was distributed in tissue culture flasks with chemically defined culture medium for human mesenchymal cells (MSCGM-CDTM Mesenchymal Stem Cell Medium, Chemically Defined, Lonza) supplemented with 2% FBS and 100 U/ml sodium penicillin and maintained at 37 °C in a humid atmosphere with 5% CO_2_. The medium was changed every 3 days until the monolayer reached confluence. The monolayer was harvested from the culture plastic with 0.125% trypsin (Gibco) and 0.78 mM ethylenediamine tetraacetic acid (Gibco). Subsequently, ASCs were analyzed by flow cytometry or differentiated for adipogenic pathway for secretory and qPCR analyses.

### Flow cytometry

Flow cytometric was performed on SVF and ASCs isolated from sSAT, sRC and dSAT. For this, cell suspension derived from SVF and ASCs was first washed once with PBS and centrifuged for 5 min at 700 g and 900 g, respectively. Subsequently, the pellet was resuspended in PBS to wash the cells and centrifuged again to obtain the pellet ready for labeling with antibodies. For immunophenotyping, the pellet was incubated for 20 min at 4 °C with monoclonal antibodies (BD Biosciences) conjugated to fluorochromes as follows: CD31 FITC, CD105 FITC, CD73 PE, CD146 PE, CD90 PE, CD34 PerCP, CD45 PE-CY7, CD34 APC. All incubations were performed in the absence of light, and then cells were centrifuged and resuspended in PBS. Incubation with FACS lysing solution (BD Biosciences) at room temperature in the absence of light for 10 min was performed on the SVF cell suspensions. For each tube, 100,000 events of SVF and 20,000 events of ASCs, respectively, were acquired in a BDFACSAria III (BD Biosciences). The flow cytometry analyses were performed using the program FACSDiva 8.0 (BD Biosciences). SVF populations have been analyzed as previously described [18]. We performed three independent experiments (*n* = 3) of six donors (*n* = 6) and three donors (*n* = 3) in SVF and ASC samples, respectively.

### Adipogenic assay

ASCs derived from sSAT, sRC and dSAT were plated on cell culture plates (2X10^4^ cells/well in a 48-well plate) or bottles (4.5 × 10^5^ cells in 75cm^2^ cell culture bottles) and subsequently exposed to the adipogenic induction medium. The induction medium is composed of DMEM with 10% FBS, 10 μM insulin, 0.5 mM isobutylmethylxanthine, 1 μM dexamethasone, and 200 μM indomethacin (Sigma). After 3 days of induction, the medium was changed, and the monolayer was maintained in a medium containing only DMEM with 10% FBS (Fetal Bovine Serum) and 10 μM insulin for additional 3 weeks. We performed four independent experiments (*n* = 4) in triplicate from four donors (*n* = 4).

### Nile Red O staining and quantification of lipid accumulation

At 3 weeks of adipogenic induction, ASC monolayer derived from sSAT, sRC and dSAT microenvironments was fixed in 4% of paraformaldehyde for 1 h at room temperature. ASC monolayer was stained with 1 mg/mL Nile Red (Sigma) diluted in 1:50 with PBS. Overstaining was washed with buffered phosphate saline. The laser was stimulated in the range of 640–720 nm. Nuclear labeling was performed with 0.5 μg/mL Hoechst. Images were acquired using a fluorescence microscope (Leica DMI 6000B) (Mannheim, Germany) equipped with Lass AF software (Leica, Mannheim, Germany). For quantification, 15 random areas were analyzed, followed by computer image analysis using ImageJ software 1.52D (NIH software, Bethesda, MD, USA, available in https://imagej.nih.gov/ij). To measure the positive Nile Red O stained area, each image was submitted to the command “Threshold.” The delimitation was adjusted by the operator in each image. After the adjustment, the “Measure” command was applied to register the percentage of the stained area. The Nile Red O positive cells count was made manually, distinguishing multilocular cells (with multiple intracytoplasmic lipid accumulation) and unilocular cells (with a single intracytoplasmic lipid accumulation) or undifferentiated cells (without  intracytoplasmic lipid accumulation). We performed four independent experiments (*n* = 4) in triplicate from two donors (*n* = 2).

### RNA isolation, quantification and quantitative real-time PCR (qPCR)

The expression levels of PPARƳ, FABP4, CEBPα and AdipoR1 genes were evaluated at 3 weeks of adipogenic induction in ASCs monolayer (75 cm^2^ cell culture bottles) derived from sSAT, sRC and dSAT microenvironments by quantitative polymerase chain reaction (qPCR). RNA extraction was performed with RNeasy Mini Kit according to manufacturer’s instructions (Qiagen, Sweden). Quantitation of mRNA was performed from the AgPath-ID™ one-step RT-PCR kit (Applied Biosystems, USA) using Applied Biosystems 7500 Real-Time PCR equipment. In summary, 1.5 μl total RNA (15 ng/μl) and amplified master mix composed of 5 μL of 2 × RT-PCR buffer, 0.4 μL of 25 × RT-PCR enzyme mix, 0.67 μl of detection enhancer and the master mix solution were rinsed with RNase free water to a final volume of 10 μL of the mixture. Specific primers and specific TaqMan probes (Applied Biosystems, USA) were used. The analyzed genes and the sequences used were as follows: PPARƳ: 5′ TCCGAGGGCCAAGGCTTCAT 3′ (Forward) and 5′ GCAAACCTGGGCGGTCTCCA 3′ (Reverse) (HS01115513_m1); FABP4: 5′ CATCAGTGTGAATGGGGATG 3′ (Forward) and 5′ GTGGAAGTGACGCCTTTCAT 3′ (Reverse) (HS01086177_m1); CEBPα: 5′ TTCACCGACAGTGGCCTTAG 3′ (Forward) and 5′ CTTTACTGCGATCGTCGTGG 3′ (Reverse) (HS00269972_m1); and AdipoR1: 5′-CCGGTTTGCCACTCCTAAGC-3′ (Forward) and 5′-TGACAAAGCCCTCAGCGATAG-3′ (Reverse) (HS00360422_m1). RPL0: 5′ CAACCCTGAAGTGCTTGACAT 3′ (Forward) and 5′ AGGCAGATGGATCAGCCA 3′ (Reverse) (Hs99999902_m1) gene expression using the ΔΔ*C*_*t*_ method. In order to compare the difference between the two conditions, the induced ASC was relativized with non-induced ASC (fold change induced versus non-induced). The expression of the non-induced ASC is shown in the graph by a dashed line. Two independent experiments (*n* = 2) were evaluated in triplicate for each gene from one donor (*n* = 1).

### Cytometric Bead Array (CBA) flex set assay

ASCs derived from sSAT, sRC and dSAT microenvironments were seeded in a 48-well plate. At 3 weeks of adipogenic induction, the culture medium was changed and after 24 h of culture medium change, the supernatant of all samples was collected and frozen at − 80 °C. The determination of proteins in the supernatant was carried out using the Cytometric Bead Array (CBA) technology (BD™) for recognition of human vascular endothelial growth factor (VEGF), interleukin-6 (IL-6) and Chemokine (C–C motif) ligand 5 (CCL5) according to the manufacturer’s instructions. The data were acquired in FACSAria III (BD Biosciences) and analyzed using software FACSDiva 8.0 and FCPA Array (3.0) (BD Biosciences). Five replicates for two independent experiments (*n* = 2) were analyzed from three donors (*n* = 3).

### Statistical analysis

Nonparametric one-way analysis of variance test followed by Dunn’s multiple comparisons test was used in order to compare data between sSAT, sRC and dSAT inside control or induced group in flow cytometry from SVF and ASCs, quantification of unilocular, multilocular and undifferentiated cells and CBA analysis. Student’s t test was used to compare the control and induced experimental conditions of ASCs from sSAT, sRC and dSAT. D'Agostino & Pearson omnibus normality test and Shapiro–Wilk normality test revealed that the data on the percentage of lipid accumulation showed normal distribution. One-way analysis of variance test followed by Sidak’s multiple comparisons test was used in order to compare data between SAT, sRC and DAT. Two-way analysis of variance test followed by Tukey’s multiple comparisons test was used in order to compare data between sSAT, sRC and dSAT in qPCR analysis.

## Results

### Pre-adipocytes dwell more frequently at the sSAT microenvironment.

**Fig. 2:**
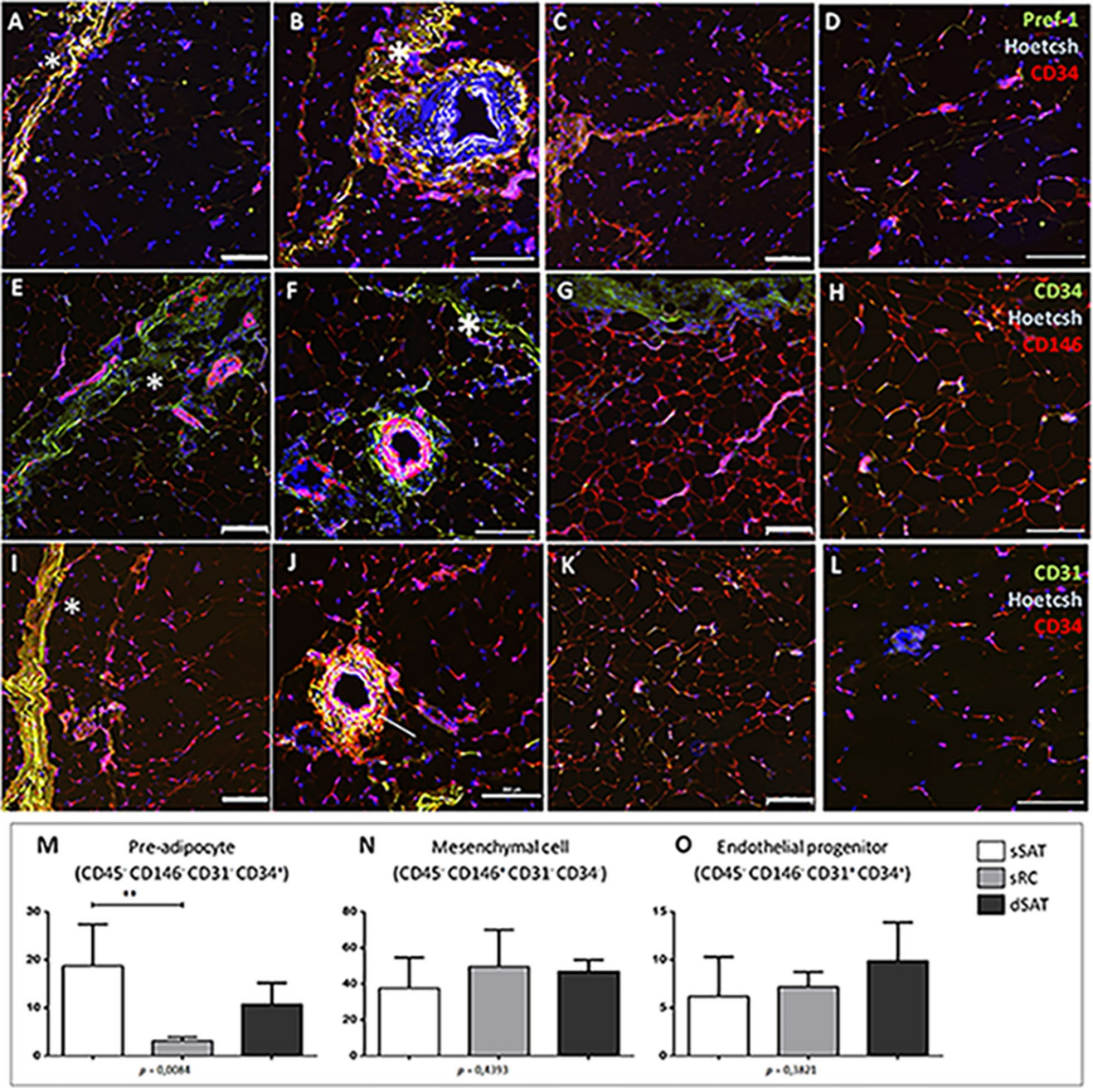
Pre-adipocytes are enriched in the sSAT layer. **A**, **B**, **E**, **F**, **I**, **J** Representative images of immunofluorescence of the sSAT microenvironment showing robust blood vessels and the sRC (asterisks). **C**, **D**, **G**, **H**, **K**, **L** Representative images of the dSAT microenvironment. **A**–**D** The Pref-1, a late marker for pre-adipocytes, was detected only in the sRC and blood vessels. Representative images showing the presence of the early marker for pre-adipocytes and endothelial progenitor cells CD34 (red in *A*–**D** and green in **E**–**H**) in the sRC, adipose tissue and blood vessels. **E**–**H** CD146, an early marker of mesenchymal stromal/stem cells, is presented at blood vessels and adipose tissue. **I**–**L** CD31, a late marker for endothelial cells, can be found associated with blood vessels as expected and to the sRC. **J** Note the presence of double positive cells (CD34/CD31, arrow). Bar size: 200 μm. Flow cytometric analysis of SVF revealed that the content of pre-adipocytes is higher in sSAT microenvironment compared with sRC (**M**). **M**–**O** The graphs represent the mean ± standard deviation of the percentage of cells analyzed in a total of 100.000 events. ANOVA test evaluated the difference between sSAT, sRC and dSAT. *p* value is described below each graph. Asterisks indicate *p* values obtained in the post-test (*p < 0.05). *SVF* vascular stromal fraction, *CD* cluster of differentiation, *SAT* subcutaneous adipose tissue, *sSAT* subcutaneous adipose tissue, *sRC* retinaculum cutis, *dSAT* deep SAT

In situ immunofluorescence for CD34, an early marker of pre-adipocytes in SAT, revealed a preferred location of these cell population at the sSAT and sRC microenvironments. In the sSAT microenvironment, pre-adipocytes are mainly located at the adventitia of robust blood vessels (Fig. 2B, F), as already described [19]. The Pref-1, a late pre-adipocyte marker [2], was co-located with CD34 at sRC and robust blood vessels of sSAT microenvironments (Fig. 2A, B). CD146, a typical marker for mesenchymal lineages, was located at robust blood vessels [19] and detected similarly at sSAT and dSAT microenvironments (Fig. 2E–H). CD31, a late marker of endothelial cells, was preferred located at the periphery of sRC and robust blood vessels, as expected (Fig. 2I, J). At the vicinity of arterioles of sSAT microenvironment, we detect a co-localization of CD34 and CD31 (yellow, Fig. 2J) representing the endothelial progenitor cells population.

To evaluate the SVF content in the three distincts microenvironments of SAT, cell suspension derived from sSAT, sRC and dSAT was monitored for surface marker expression. The percentage of pre-adipocytes in sSAT was significantly higher compared to RC (*p* = 0.0212) (Fig. 2M), supporting immunofluorescence images.

### ASCs derived from sSAT, sRC and dSAT microenvironments showed remarkable differences in their adipogenic potential

**Fig. 3:**
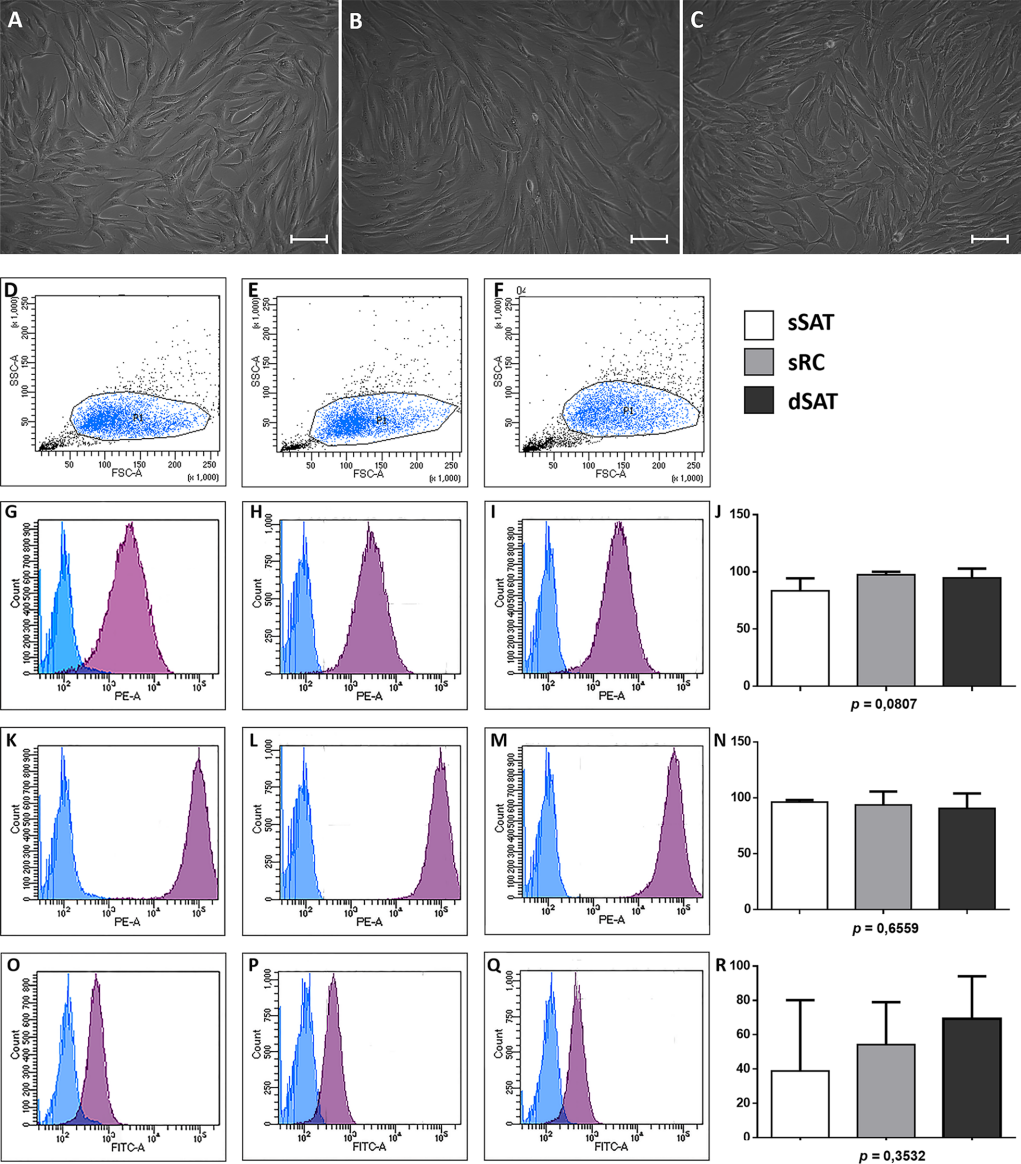
ASCs from sSAT, sRC and dSAT showed similar morphology and mesenchymal stromal/stem cells surface marker. **A**–**C** ASCs from sSAT, sRC and dSAT microenvironments showed a similar fibroblastic morphology. **D**–**F** Cells of each adipose tissue depot are shown in a forward versus side scatter dot plot. **G**–**I** Representative histogram of the surface marker CD73; **K**–**M** CD90 and **O**–**Q** CD105. Blue histograms represent the unstained cells, and the purple histograms represent the stained (positive) cells. **J**, **N**, **R** The graphs represent the mean ± standard deviation of the percentage of positive cells. ANOVA test evaluated the difference among sSAT, sRC and dSAT microenvironments. *p* value is described below each graph. *Count* event count, *ASCs* adipose stromal/stem cells, *CD* cluster of differentiation, *SAT* subcutaneous adipose tissue, *sSAT* superficial SAT, *sRC* retinacula cutis superficialis, *dSAT* deep SAT

ASCs derived from sSAT, sRC and dSAT microenvironments showed similar fibroblastic morphology (Fig. 3A–C) and mesenchymal cell surface markers expression (Fig. 3G–R).

**Fig. 4:**
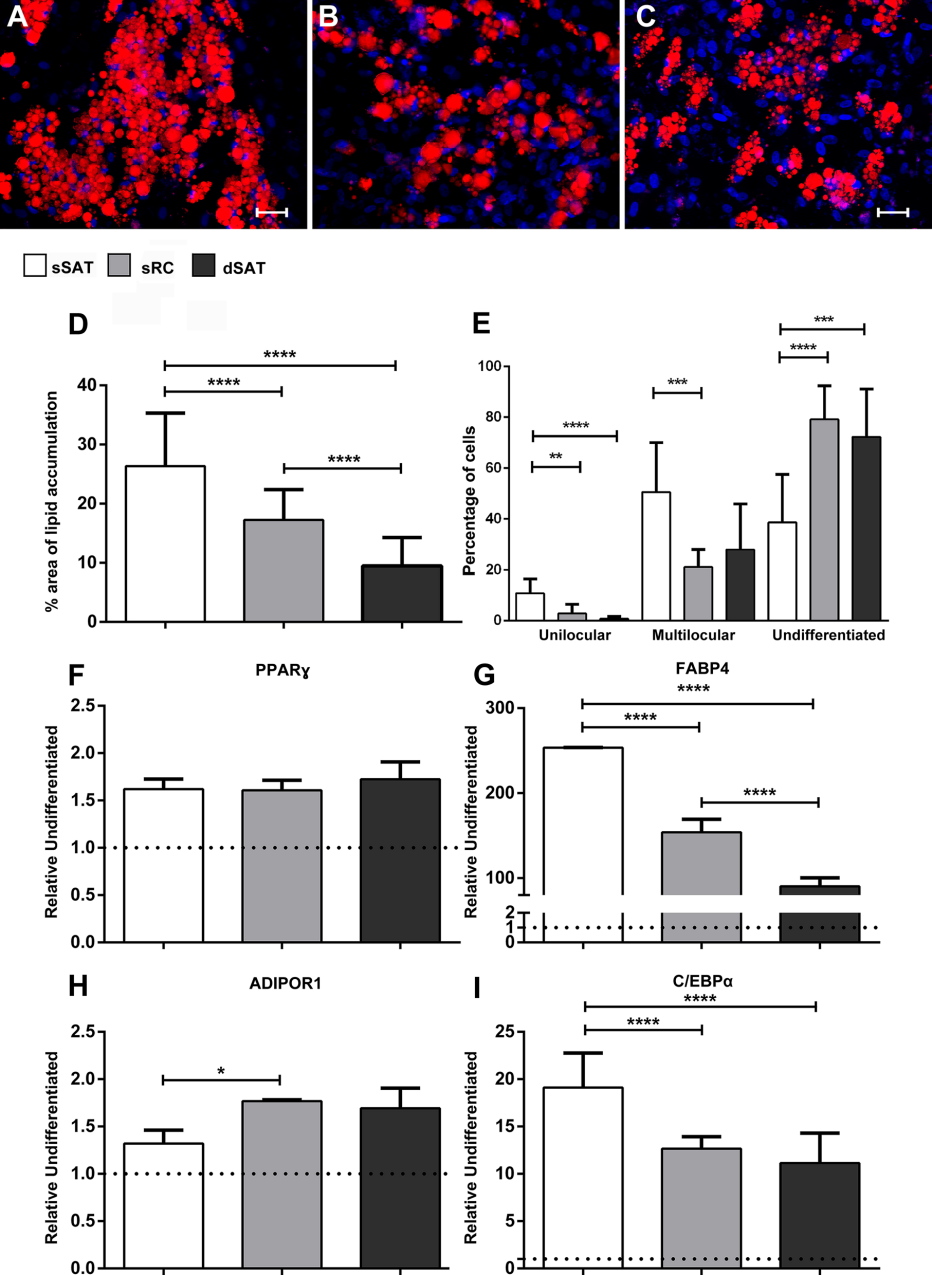
ASCs derived from sSAT microenvironment showed an accelerated adipogenesis compared with sRC and dSAT. **A**–**C** Representative images of adipogenic-induced ASCs derived from sSAT, sRC and dSAT microenvironments. The intracytoplasmic lipid accumulation were revealed by Nile Red O staining (red) and the nuclei by Hoechst (blue). Bar size: 50 μm. **D**, **E** The percentage of the area of lipid accumulation and the percentage of unilocular, multilocular and undifferentiated cells are expressed in the graphs as mean ± standard deviation. ASCs derived from sSAT showed the highest area of lipid accumulation together with the highest percentage of unilocular cells. **F**–**I** qPCR analysis of ASCs derived from sSAT, sRC and dSAT microenvironments revealed an upregulation of all evaluated genes in adipogenic-induced ASCs compared with non-induced. **F** PPARgamma; **G** FABP4; **H** ADIPOR1; **I** CEBPα. The gene expression of adipogenic-induced ASCs was relativized to the gene expression of non-induced (dashed line). The graphs represent the mean ± standard deviation. ANOVA test evaluated the difference between sSAT, sRC and dSAT microenvironments. *p* value is described below each graph. Asterisks indicate *p* values obtained in the post-test (*p < 0.05; **p < 0.001; ***p < 0.0005; ****p < 0.0001). *ASCs* adipose stromal/stem cells, *SAT* subcutaneous adipose tissue, *sSAT* superficial SAT, *sRC* retinacula cutis superficialis, *dSAT* deep SAT

ASCs derived from sSAT microenvironment were able to accumulate a higher area of intracytoplasmic lipids compared with sRC (*p* < 0.0001) and dSAT (*p* < 0.0001) (Fig. 4A–E). Furthermore, sRC presented higher intracytoplasmic lipid accumulation compared with dSAT (*p* < 0.0001) (Fig. 4D). ASCs derived from sSAT microenvironment showed a higher percentage of cells at the unilocular stage compared with sRC (*p* = 0.0024) and dSAT (*p* < 0.0001) and a higher percentage of cells at the multilocular stage compared with sRC (*p* = 0.0002) (Fig. 4E). The analysis of genes involved in adipogenesis revealed no significant differences in the levels of PPARγ, a master gene of adipogenesis [20, 21] among the different sources of ASCs. As expected, we identified an upregulation in all experimental groups of adipogenic-induced ASCs compared with non-induced: sSAT (*p* = 0.0001) sRC (*p* = 0.0002) and dSAT (*p* =  < 0.0001) (Fig. 4F). The FABP4 gene, involved in the expression of metabolic genes and adipokines [22, 23], showed significant differences in adipogenic-induced ASCs from sSAT microenvironment compared with sRC (*p* < 0.0001) and dSAT (*p* < 0.0001). Adipogenic-induced ASCs from dSAT microenvironment showed the lowest level for FABP4 gene (Fig. 4G). The hormone adiponectin is considered as having anti-inflammatory properties [24]. In this study, gene levels of its receptor adiponectin receptor 1 (ADIPOR1) were analyzed. Adipogenic-induced ASCs from sRC microenvironment showed a significant increase in this gene level (*p* = 0.0409) compared with adipogenic-induced ASCs from sSAT (Fig. 4H). In a similar way to FABP4 gene, C/EBPα (CCAAT/enhancer-binding protein alpha) gene showed significant high levels in adipogenic-induced ASCs from sSAT microenvironment compared with sRC (*p* < 0.0001) and dSAT (*p* < 0.0001) (Fig. 4I). The CEBPα gene belongs to the family of adipogenic regulators together with the PPARγ gene [23].

### Adipogenic-induced ASCs from sRC microenvironment showed the highest level of growth factors and cytokines

**Fig. 5:**
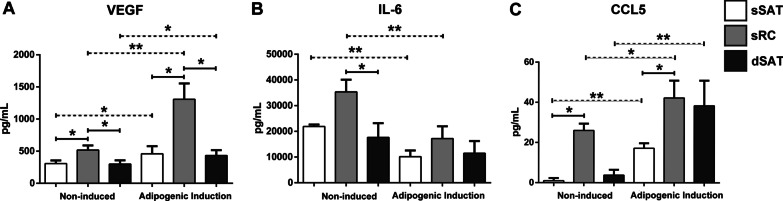
ASCs derived from sRC microenvironment showed the highest level of the chemokine CCL5. **A** VEGF; **B** IL-6 and **C** CCL5. The graphs represent the mean ± standard deviation. ANOVA test evaluated the difference between sSAT, sRC and dSAT microenvironments within each non-induced and induced group. Continuous lines indicate post-test analyses within both conditions. Dashed lines indicate t test analyses, which was performed in order to verify the statistical difference between the non-induced and induced group of the sSAT, sRC and dSAT microenvironments. Asterisks indicate *p* values obtained in the post-test and in the post-test and in the t test (*p < 0.05; **p < 0.001). *ASCs* adipose stromal/stem cells, *SAT* subcutaneous adipose tissue; retinaculum cutis, *sSAT* superficial SAT, *sRC* superficial, *dSAT* deep SAT, *VEGF* vascular endothelial growth factor, *IL* Interleukin, *CCL5* chemokine (C–C motif) ligand 5

The secreted profile of soluble mediators by adipogenic-induced and non-induced ASCs from sSAT, sRC and dSAT microenvironments was evaluated. The level of VEGF was significant high in non-induced ASCs from sRC compared with sSAT (*p* = 0.0485) and dSAT (*p* = 0.0112) and induced ASCs from sRC compared with sSAT (*p* = 0.0175) and dSAT microenvironments (*p* = 0.0328) (Fig. 5A). For interleukin-6 (IL-6), we observed a significant decrease (*p* = 0.0079) after adipogenic induction in ASCs from sSAT (Fig. 5B) and sRC (*p* = 0.0079) microenvironments. Non-induced ASCs from sRC showed the highest level for CCL5 (Chemokine (C–C motif) ligand 5) compared with non-induced (*p* = 0.0029) and induced ASCs from sSAT microenvironment (*p* = 0.0089) (Fig. 5C). CCL5 is a potent chemoattractant for immune cells also having an important role as an angiogenic and migration factor in ASCs [25, 26].

## Discussion

The present study analyzed the SVF content exploiting their cell populations more broadly than previous studies which analyzed the sSAT and dSAT microenvironments. Furthermore, we deciphered ASC behavior depending on their superficial or deep location and unveiled a conjunctive derived microenvironment: the sRC. The present study revealed the importance within the sSAT of the sRC with angiogenic potential.

The analysis of SVF content using in situ immunofluorescence and ex vivo flow cytometry revealed that mesenchymal stromal/stem cells are located in all the distinct SAT microenvironments. Pre-adipocytes are preferentially located in sSAT and sRC, while endothelial progenitor cells in sSAT are mainly located at the vicinity of sRC and blood vessels. sSAT microenvironment revealed rather robust vessels containing the conjunctival layer (adventitia) with pre-adipocytes showing positivity for CD34 and Pref-1. Pref-1 is a cell surface marker mainly involved in maintaining pre-adipocyte phenotype [27–29], having been observed only in sSAT and sRC. Together these results support the sSAT microenvironment as hyperplastic due to its enrichment in pre-adipocytes.

The analyses of adipogenic potential of ASC derived from the three different microenvironments also support the hyperplastic phenotype of the sSAT. We found a significantly greater area of intracytoplasmic lipid accumulation in ASCs induced to adipogenic lineage from the sSAT microenvironment together with the highest number of unilocular cells. Kosaka and collaborators showed that adipocytes from the sSAT occupy a greater area compared with the dSAT, besides having a higher quantity of these cells [12]. Previous studies have shown a higher adipogenic potential from sSAT compared with dSAT isolated from obese samples [11, 13, 30]. Our study relies on SAT samples isolated from individuals showing different BMI (non-obese ranges) and nationalities offering a more real insight into the contribution of these distinct microenvironments to the physiology of SAT. Furthermore, few studies related to sSAT and dSAT microenvironments have analyzed adipogenesis from ASCs [10, 12, 30].

ASCs induced to adipogenic lineage from sSAT, sRC and dSAT microenvironments showed a significant increase in PPRAɣ gene expression compared with non-induced. The PPARɣ gene, a master regulator of adipogenic differentiation, has been reported as having a higher expression in SVF of sSAT microenvironment compared with dSAT in non-obese [12] and obese samples [13]. The CEBPα gene drives differentiation by up-regulation of adipogenic genes. Although we found no differences in PPARγ gene expression, CEBPα gene is upregulated in ASCs induced to adipogenic lineage from sSAT compared with sRC and dSAT microenvironments. Kosaka and collaborators observed a high CEBPα gene expression in SVF of dSAT microenvironment [12]. The discrepancies among our results from others probably rely on the heterogeneity of the SVF analyzed by Kosaka and Cancello. The SVF contains cells in different stages of differentiation besides hematopoietic lineage, including macrophages.

Recently, Cappellano and collaborators showed a higher adipogenic potential of ASCs derived from sSAT compared with dSAT microenvironment, supporting our results [30]. The fatty acid binding protein—FABP4 [31, 32] is one of the most expressed proteins in mature adipocytes [12]. According to the highest number of unilocular cells in ASCs from sSAT, we found a significant upregulation of FABP4 gene expression on ASCs induced to adipogenic lineage from sSAT compared with sRC and dSAT microenvironments. All results from cells derived from sSAT in this study attested its hyperplastic phenotype: the highest expression of pre-adipocyte markers of SVF and the highest adipogenic potential of ASC.

In humans, the sRC represents extensions from superficial fascia lying in SAT. In rats, the superficial fascia contains adipocyte progenitor cells [2] being a tissue microenvironment distinct from the dermis and the SAT. Recently, Zhang and collaborators have observed subtle differences in adipocytes isolated from rat superficial fascia compared with adipocytes from visceral and SAT [33]. In this study, adipogenic induction revealed a lower adipogenic potential from ASCs derived from sRC compared with the sSAT microenvironment. The hyperplastic phenotype of sSAT observed by previous studies [11, 12, 14] was evaluated in the presence of sRC. In this study, sSAT was analyzed ex vivo without the sRC content. Based on this, we can consider that the hyperplastic phenotype of sSAT in humans is derived only from its cell content.

Curiously, the ADIPOR1 gene was significantly upregulated in ASCs induced to adipogenic lineage from sRC compared with sSAT. The dSAT microenvironment is commonly described as having a pro-inflammatory profile [10, 34]. On the other hand, the sSAT microenvironment is described as an anti-inflammatory profile. Both inflammatory profiles are not explored in this study. Marinou and collaborators analyzed the whole SAT microenvironments showing a higher expression of ADIPOR1 gene in sSAT compared with dSAT [34]. We can postulate that the sRC could influence the anti-inflammatory profile from the sSAT microenvironment. Additional experiments are in progress to unveil sRC inflammatory role in SAT.

ASCs induced and non-induced to adipogenic lineage from sRC showed the highest levels for secreted molecules analyzed in this study. In the recent study of Su and collaborators [2], the authors claimed that once SAT is formed, this fascia remains as a connective tissue that tightly interdigitates the whole tissue. In this study, the loose connective tissue represented by sRC was dissected only from the sSAT microenvironment. ASCs from sRC secreted higher levels of VEGF compared with sSAT and dSAT microenvironments. Di Taranto et al. [14] reported a higher VEGFA gene expression from sSAT, including sRC, making it difficult to determine which SAT microenvironment substantially contributes to angiogenesis. Based on our results, we believe that ASCs from sRC are responsible for the angiogenic capacity of superficial SAT. Besides, in humans, cells derived from RC may account for vasculogenesis during SAT formation in developing embryos.

The secretion of the chemokine, CCL5, was significantly higher on ASCs from sRC compared with sSAT microenvironment. CCL5 was originally described as a pro-inflammatory cytokine [35–37], and recently, as having a crucial role in angiogenesis of SAT [37–39]. The highest secretion of CCL5 on ASCs from sRC supports their secretion profile for VEGF. Curiously, the secretion of IL-6 was higher in non-induced ASCs from sRC compared with dSAT. In a pathological condition such as obesity, dSAT influences the sSAT, leading to cellular and molecular alterations [13, 30]. In the same way, sRC could influence sSAT since it represents a SAT microenvironment by itself.

## Conclusions

Human SAT should be considered as three functionally distinct microenvironments rather than a single entity. In humans, as described previously in rats, the superficial fascia gives origin to the RC in superficial and deeper layers, representing a mesenchymal microenvironment distinct from sSAT and dSAT. Additional studies are mandatory to understand the true pleiotropic angiogenic role of sRC and its influence in sSAT during healthy and pathological conditions. Furthermore, the local injection of ASCs derived from RC could be used aiming for a structural restoration of skin flaccidity and for wound healing due their angiogenic capacity.

## Data Availability

All data generated or analyzed during this study are included in this published.

## Abbreviations

SAT: Subcutaneous adipose tissue
Ssat: Superficial SAT
dSAT: Deep DAT
RC: Retinaculum cutis
SVF: Stromal-vascular fraction
ASCs: Adipose-derived stromal/stem cells
sRC: Superficial RC
PBS: Phosphate buffered saline
PBS-T: Triton X-100
BSA: Bovine serum albumin
MSCGM-CDTM: Chemically defined culture medium for human mesenchymal cells
qPCR: Polymerase chain reaction
CBA: Cytometric Bead Array
VEGF: Vascular endothelial growth factor
FABP4: Adipocyte fatty acid-binding protein
IL-6: Interleukin-6
CCL5: Chemokine (C–C motif) ligand 5
FAPERJ: Carlos Chagas Filho Foundation for Research Support of the State of Rio de Janeiro
